# Supplementary material for: Are carbon emissions trading and green financial instruments synergistic? -Comprehensive quantitative research based on content analysis
Source: PLoS One. 2024 Mar 7;19(3):e0298601. doi: 10.1371/journal.pone.0298601 (PMC10919601; doi:10.1371/journal.pone.0298601)
Supplement: S1 Data — (ZIP) [file pone.0298601.s001.zip › Data/地级市Z_F工作报告.zip!/╔╜╬≈/┤≤═1⁄4╩╨/└┤╫╘╣1⁄2╓┌║┼í╛┬φ┐╦╩2╛▌═°í┐_2019.docx]

各位代表:

现在,我代表市人民政府,向大会报告工作,请予审议,并请市政协委员和其他列席人员提出意见。

一、2018年工作回顾

过去的一年,全市上下高举习近平新时代中国特色社会主义思想伟大旗帜,认真贯彻落实党的十九大精神和习近平总书记视察山西重要讲话精神,抢抓经济转型的重要窗口期、攻坚期,坚定战略自信,保持战略定力,以“示范区”“排头兵”“新高地”三大目标为牵引,坚定不移实施“136”发展战略,加快创新驱动转型升级步伐,扎实推进新旧动能转换,着力推动高质量发展,圆满完成了各项目标任务,为全面建成小康社会奠定了坚实基础。

——综合实力在攻坚克难中实现新跨越。针对经济运行稳中有变、下行压力加大的形势,全力打好稳增长组合拳,多项指标争先进位,跃升到合理区间。地区生产总值完成1271.8亿元,增长6.8%,增速排名全省第6;规模以上工业增加值完成334.9亿元,增长5.7%,增速排名全省第6;固定资产投资完成549.2亿元,增长12.3%,增速排名全省第3;第三产业增加值完成742.8亿元,增长8.2%,增速排名全省第7;公共财政预算收入完成119.7亿元,增长10.5%,增速排名全省末位;社会消费品零售总额完成700亿元,增长8.1%,增速排名全省第6;城乡常住居民人均可支配收入分别达到29911元和9710元,增长6.9%和9.6%,增速均排名全省第2;外贸进出口总额完成37.56亿元,增长52.5%,增速排名全省第2。

——能源革命在“尖兵”实践中取得新突破。坚持把能源革命作为大同高质量发展的最大王牌和经济转型发展的最亮品牌,下大气力推动平台搭建、人才聚集、项目建设、机制保障、科技成果落地和宣传发动,形成全面发力、多点突破的工作格局。能源革命科技创新园高位起步,“氢都”新能源产业城开工建设。迅速引进全科盟、中科院10所研究所,成立新能源产业局、新能源产业战略咨询委员会、全科盟新能源产业技术研究院,国内外高端能源研发智库汇聚大同。21名两院院士、71名能源领域领军人才竞相集聚大同,零排放煤化工、燃气热泵产业化等7项重大科技成果转化落地。北京汉能、西安隆基、深圳雄韬等能源革命十大突破工程稳步推进。成功举办首届“能源革命、对外开放”科技周国际高峰论坛,在“能源1号”举办能源革命百家讲座91讲,在大同日报每天整个版面、大同电视台每天新闻之后半个小时,开辟专栏专题进行能源革命讲座宣传。在能源革命交易博览中心和科创园新产品展示中心举办119项新能源新材料展览100余场次,积极抢占央视、高速、高铁、机场等重要平台宣传能源革命、开展项目招商,“争当能源革命尖兵、走出大同转型新路”已经成为大同最亮的一张名片,“能源革命·大同尖兵”已经成为大同人民合力奏唱的一曲新时代最亮的高歌!

——改革开放在全面深化中迈出新步伐。实施走出去战略,设立京津冀、长三角、珠三角、雄安新区、一带一路、以色列特拉维夫六大高科技产业项目处,与国家商务部投促局、上海漕河泾开发区、中关村智造大街建立常态化对接合作机制,建立中关村智造大街大同服务平台和大同企业离岸创新孵化基地北京站,与北京海淀区、丰台区、上海松江区、华东理工大环境工程学院、南方科大创新创业学院、复旦大学生命工程学院等区校签订了战略合作协议,着力搭建产业合作新平台。启动谋划京晋(大同)协作新区和魏都新区,打造对外开放新窗口。在北京、深圳、西安、苏州成功举办大同“氢都”新能源产业城招商推介会,新引进项目139个、总投资1200亿元。坚持古城区、御东新区、同煤新区、开发区“四区”联动发展战略,百亿城建工程圆满收官,开源桥建成通车,永泰南门广场主体完工,云冈机场跻身全国百万级机场行列,同煤至云冈机场快速干线加快实施,大西、大乌高铁列入国家“八横八纵”交通网络,对外开放城市形象得到全面提升。行政区划调整圆满完成,重点领域和关键环节改革持续深化。

——产业结构在强力反转中呈现新趋势。大抓、狠抓、强抓、实抓工业振兴,全市规模以上工业总产值突破千亿元大关。非煤工业增加值增长14.5%,占规模以上工业增加值比重达到56.1%;制造业增加值占规模以上工业增加值比重上升3.5个百分点;战略性新兴产业、高技术产业增速分别达到28.1%和28.5%,占到规模以上工业增加值的比重达到17.5%和8.5%。大大快于规模工业平均增速。深入开展转型项目建设年活动,全年开工建设的176个工业转型大项目建成99个,墨西科技年产10吨石墨烯粉体、雄韬年产1万套氢燃料电池发动机总成、中银纺织2条洗毛生产线、西安隆基单晶硅电池组件、晋投玄武岩等一批高端项目相继投产。出台鼓励和支持民营企业发展的“二十条”政策措施,拿出5亿元工业振兴奖励专项资金,对同煤集团、同车公司、宇林德石墨、新成新材料等贡献突出的企业进行了大奖励、硬扶持。坚持把文旅产业作为资源型经济转型的主攻方向,加快全域旅游示范区创建,大手笔运作古都灯会和成龙国际动作电影周,潘家园古玩市场落成运营,“火山天路”、古长城旅游路等项目扎实推进,大同文旅正在成为全国闻名、世界瞩目的旅游品牌。全市旅游总收入达到620.9亿元,同比增长28.5%。三次产业结构调整为5.1∶36.5∶58.4。

——农业农村工作在强基固本中达到新水平。粮食总产量达到24.6亿斤,实现“九连丰”。“一带三区”建设扎实推进,粮改饲、设施农业、标准化养殖、有机旱作农业加快发展。拿出10亿元专项资金,大力实施乡村环境提升工程,安家皂、涧西等102个示范村实现村容村貌、户容户貌整治全覆盖。与北京首农、广东农垦、中国农大、中国农科院、山西农大、京东集团等名企名校开展战略合作,“大同好粮”正在阔步走上特色化、品牌化、市场化的高效发展之路。聚焦深度贫困,全面压实责任,对标“六个精准”,落实“五个一批”,“一县一策”,精准滴灌,用心用情用力抓脱贫。云州黄花特色产业扶贫、灵丘资产收益扶贫、阳高集中式搬迁扶贫等工作走在全省前列,3个贫困县区、264个贫困村、10.4万贫困群众实现了脱贫摘帽,全市贫困发生率降至1.78%,我们正在大步走上全面建成小康社会的康庄大道!

——生态环境在标本兼治中得到新改善。综合施策提升“大同蓝”,严格划定城市禁煤区,依法淘汰燃煤锅炉和柴油机车,新增及更换新能源或清洁能源公交车比重达到75%,持续扩大城市集中供热覆盖面,取缔“散乱污”企业209家,全市二级以上优良天数比例达到83.7%,全省排名第1,从“煤都黑”到“大同蓝”的典型经验和做法受到国务院通报表扬;强力保障“大同清”,大力实施桑干河流域五大污水处理厂提标改造工程,集中开展河道清淤清障,持续加大黄河生态补水增量,全面加快御河生态湿地修复,水生态环境显著改善;持续厚植“大同绿”,着力打造环首都生态安全屏障,完成营造林19.57万亩,全市建成区新增绿化面积165.7万平方米、绿化覆盖率达到42.87%、绿地率达到38.75%、人均公园绿地面积达到15.88平方米,美丽大同建设卓有成效,“大同蓝”成为大同生态环保建设一道新的靓丽的风景线!

——民生事业在成果普惠中取得新进步。全市财政民生支出占比达到81.5%。教育事业加快发展,普惠性幼儿园覆盖率达到86.7%,县域义务教育均衡发展全部高质量通过国家复评。全面改薄累计投入资金8.39亿元,城乡中小学办学条件显著改善。县管校聘改革成效显著,高中教育办学水平正在加速提升。省教育厅与大同市共建大学模式取得重大进展。文化体育事业成绩斐然,传统文化村落和非物质文化遗产保护工作持续加强,春节文化庙会和民俗大展演等文化活动普惠群众,群众性文体活动蓬勃开展。大同图书馆、市少儿图书馆、市群众艺术馆免费开放,“书香大同”赢得真情点赞。全国第二届青年运动会冬季项目成功举办。千方百计拓宽就业渠道,就业创业形势喜人,超额完成年度目标任务。医疗卫生事业长足进步,公立医院改革持续深化,市五医院等三大医疗集团正式挂牌成立;在全省首家实行药品二次议价,年可节约医保资金和患者个人费用2亿元;分级诊疗、医养结合等便民医疗改革扎实推进。社会保障体系更加完善,落实社会救助资金10.8亿元,发放各类低保金6.7亿元,农村低保标准高于全省平均水平。在9个农业县区建立“孝养基金”试点,成功列入全国第三批社区和居家养老服务试点城市。安居工程扎实推进,全市棚改项目开工8806套,基本建成22772套,公租房建设完成2810套,完成投资27.29亿元。下大气力解决国有土地上房屋权属登记历史遗留问题,分8批办理了102个小区的房屋权属登记工作。平安大同建设成效显著,全面创新社会治理体系,狠抓基础能力建设,安全生产形势平稳向好。治超工作实现由奋力赶超到引领全省的根本转变。防震减灾工作荣获国家级表彰。扎实开展扫黑除恶专项斗争,成功打掉涉黑涉恶犯罪团伙106个、全省排名第二。市政府年初确定的10件惠民实事全部兑现,实实在在地提高了人民群众的获得感、幸福感、安全感。

——政府自身建设在服务升级中迈上新台阶。将学习贯彻习近平新时代中国特色社会主义思想贯穿于政府工作全过程,推动习近平总书记视察山西重要讲话精神提到的“五项重大任务”在大同变成生动实践。认真落实党风廉政主体责任,真管真严、敢管敢严、长管长严,将全面从严治党要求落到工作的每一个角落。始终坚持依法行政,办理人大代表建议193件、政协委员提案300件,办结率均为100%。主动接受监委监督,实现对所有行使公权力的公职人员监察监督全覆盖。在全市政府系统开展干部素质能力大提升活动,干部推动发展、解决矛盾、服务群众的能力显著增强。持之以恒转变作风,严格执行中央八项规定精神,自觉践行省委“五倡导五反对”,激励引导广大干部激情干事、开拓干事、精准干事,形成了大力度、快节奏、高效率的工作作风。在全市大力推行“送服务、送环境、送效率、送资源、送市场”的“五送”服务行动,把“六最”营商环境转化为现实生产力。与此同时,信访、统计、民族、宗教、对台、侨务、民防、气象、双拥、国防动员、档案、史志等工作取得新进步。

各位代表,过去的一年,是我们贯彻新发展理念、对标省委“三大目标定位”、综合实力实现显著提升的一年,是能源革命多点突破、对外开放多措并举、现代化经济体系加快构建的一年,是各项社会事业更加繁荣、社会治理体系更加健全、社会和谐局面更加稳固的一年,是发展理念不断创新、重点领域改革不断深化、人民群众收入不断提高的一年。这些成绩的取得,是习近平新时代中国特色社会主义思想科学指引的结果,是省委省政府和市委正确领导的结果,是市人大政协有力监督的结果,是社会各界热心支持的结果,也是全市广大干部群众戮力同心、艰苦奋斗的结果。在此,我代表市人民政府,向所有为大同改革发展作出贡献的同志们、朋友们,表示崇高的敬意和衷心的感谢!

在肯定成绩的同时,我们也要清醒地看到,政府工作还存在着一些不容忽视的问题:

一是经济运行质量不高。2018年全市地区生产总值仅占全省地区生产总值总量的7.6%,总量绝对额排在全省第8,与全省第二大城市的地位不相匹配;煤电产业仍占主导地位,高效益、高产出、高拉动型的非煤现代产业尚未形成气候,“一煤畸重”的结构性矛盾短期内难以得到有效化解;城乡常住居民人均可支配收入虽然增势强劲,但绝对额排在全省的末位,经济发展活力明显不强。

二是创新能力不足。高技术企业数量少,高精尖人才不足,资金投入不够;众创空间数量少,标准不高;企业创新意识不强,市场理念不浓,企业产品科技含量低,技术改造升级缓慢,以科技创新为驱动的产业发展模式还尚未形成;围绕服务转型、服务企业、服务发展,政府在创建“制度高地”、“政策高地”等方面工作主动性不强、创新的好办法还不多。

三是民生领域欠账较多。市本级财力对民生保障能力弱,城乡基础设施建设滞后,城市公共服务水平低,教育、卫生、社保、住房等老百姓热切关注的民生工程还存在诸多堵点、痛点,政府要完成的“作业”量大任重。

四是“三大攻坚战”面临严峻挑战。作为典型的大市小财政,既要保吃饭、又要促发展、还要积极地化解债务风险,多重压力交错叠加;深度贫困量大、面广,精准脱贫面临最难啃的硬骨头;环保约束性指标不降反升,二级以上优良天数跌破300天,污染防治还存在诸多短板。

五是发展环境亟待进一步优化。有些部门工作效率低下、标准不高、服务意识不强,少数干部不善作为、与市场打交道能力弱的问题十分突出。对此,我们要认真面对,采取有力措施加以解决。

二、2019年经济和社会发展主要任务

2019年是中华人民共和国成立70周年,是全面建成小康社会的关键之年,是我市改革开放再出发、转型发展再发力、“两个尖兵”再攻坚、综合实力再跃升的奋进之年。今年政府工作的总体要求是:以习近平新时代中国特色社会主义思想为指导,全面贯彻党的十九大和十九届二中、三中全会精神,统筹推进“五位一体”总体布局,协调推进“四个全面”战略布局,深入贯彻习近平总书记视察山西重要讲话精神,按照中央和省委经济工作会议精神,以及省委十一届六次、七次全会部署,在市委的坚强领导下,坚持稳中求进工作总基调,坚持新发展理念,坚持推动高质量发展,坚持把供给侧结构性改革与转型综改试验区建设相结合作为经济工作主线,坚持深化市场化改革、扩大高水平开放,加快建设现代化经济体系,大力实施“136”发展战略,打造转型发展先行区,奋力争当“两个尖兵”,持续推进“三大振兴”,继续打好“三大攻坚战”,大力实施“十二大工程”,扎实做好稳增长、促改革、调结构、惠民生、防风险工作,全力争取“两转”基础上的更大进步,为全面建成小康社会收官打下决定性基础,以优异成绩庆祝中华人民共和国成立70周年。

今年的主要预期目标是:地区生产总值增长7%,第三产业增加值增长7.5%,规模以上工业增加值增长6.5%,全社会固定资产投资增长10%,社会消费品零售总额增长7%,公共财政预算收入增长7.5%,城乡常住居民人均可支配收入分别增长7%以上和7.5%,对外贸易进出口总额增长5%,居民消费价格指数控制在3%左右。各项约束性指标和转型考核指标完成省下达的考核任务。

做好今年工作,我们要做到“三个必须”:

一是必须鲜明确立“大转型、大创新、大改革、大开放、大提标、大市场”六大工作要求。“大转型”就是要紧紧扭住转型升级这个核心,坚持“一切为了转型、一切聚焦转型、一切服务转型”,当好能源革命尖兵,加快工业结构性反转,横下一条心,培育新动能,全力走出大同转型新路!“大创新”就是要坚持把创新作为引领发展的第一动力,着力打造项目承接、科研推广、高端智库和成果转化平台,实现金融、技术、人才、产业、业态和体制机制创新一起上,壮大创新主体,提升创新实力,锻造“双创”筋骨,大力增强创新自信!“大改革”就是要大力开展“改革创新、奋发有为”大讨论,凝聚改革共识,争当改革尖兵,在重点领域和关键环节改革上勇于先行先试、敢闯敢试,坚决破除发展瓶颈,最大限度释放发展活力!“大开放”就是要当好对外开放“尖兵”,彻底改变眼睛向内的习惯,坚决“跳出大同看大同”,就是要坚持世界眼光、高点定位,以开放促改革,以改革促转型,全力打造融入京津冀桥头堡!“大提标”就是要“对标作战、向标看齐、用标推进、对表交卷”,样样工作争一流,拉高思想境界的标杆,前推发展理念的起跑线,争先进位,全力冲刺全省第一方阵!“大市场”就是要坚持市场引领,盯住市场需求,围绕高端制造、新能源、新材料等新兴产业,在全省全国市场中找商机、引项目、办企业,培植大同发展的后劲优势!

二是必须大力实施对接“大企大校大院大所”四大工作举措。要强化对接“大企”意识,主动对接世界500强、中国500强,紧紧围绕构建12大产业集群,挂靠大企业,实施大项目,为做强做大实体经济构建硬支撑、硬后盾!要强化对接“大校”意识,主动对接清华、北大以及国际名校,强化战略合作,对接一流名校,培养一流人才,为转型发展培育资源,积累资本!要强化对接“大院”意识,积极对接中国科学院、工程院和农科院,引进市场机制,加强科研攻关,推动成果落地,切实增强企业生产发展的最大潜力!要强化对接“大所”意识,致力转型升级,引进科研院所,攻克关键难题技术,为实现“大同制造”向“大同创造”“大同智造”转变奠定坚实基础!

三是必须坚定不移开展“送服务、送环境、送效率、送资源、送市场”“五送”服务。“大送服务”就是要主动登门、主动服务,想企业所想,急企业所急,甘当服务企业的“店小二”!“大送环境”就是要围绕发展所需,大力营造廉洁高效的政务环境、民主公正的法制环境、公平诚信的市场环境、安全稳定的社会环境。要加强政企互动,构建“亲”“清”新型政商关系,真心营造政企“一家人”环境!“大送效率”就是要持续深化“放管服效”改革,马上办、争着办、真情办,竭诚当好为企业提供服务的“快递哥”!“大送资源”就是要打破常规,与国内国际一流企业加强合作,整合市内交通、矿产、文化等各类资源,高端策划,打包运营,以资源优势换取转型发展的经济优势,大力提升大同资源附加值、增加值!“大送市场”就是要纾解企业所困,下大气力敞开能源消费、新能源汽车、智慧城市等各类市场,帮助企业做大做强,用市场容量和市场消费助推大同转型发展!

围绕上述目标和总体要求,今年要重点抓好十二大工程:

(一)能源革命重大突破工程

抢抓全省开展能源革命综合试点的重大机遇,争创全省能源革命示范市,以打造现代能源产业体系作为总体方向,重点在六个方面实现重大突破。

一是在煤炭“减、优、绿”上实现重大突破。全年压减煤炭产能135万吨;推进煤矿减量重组,淘汰落后产能630万吨。分类指导、有序推进基建矿井产能释放,建成同煤集团同忻煤矿千万吨级矿井的智能化升级项目,左云鹊山产能达到350万吨,同煤塔山、同煤同忻和中煤塔山核增先进产能2850万吨,长春兴煤矿完成二代机器人和“云控矿山”升级改造,将先进产能占比提高到72.8%。开工建设1座煤矸石填充开采示范项目。推动中海油煤制气、同煤烯烃和煤制氢三大煤化工项目开工,我们要把煤炭产业做深做长,全面开启燃料煤向原料煤转变的新时代。

二是在新能源产业上实现重大突破。建成120万千瓦晋北风电基地项目、50万千瓦国家光伏领跑者二期项目,开工建设150万千瓦光伏平价上网示范项目,将清洁能源发电装机占比提升10个百分点。着力打造新能源高端装备制造业,聚焦新能源汽车、氢能、光伏制造产业链、储能等领域,重点推进陕汽集团新增1万台天然气重卡扩建项目、深圳雄韬年产1万套氢燃料电堆发动机总成项目、北京新研年产1万套氢燃料动力系统项目、山西城市动力年产1GW储能动力电池项目、西安隆基年新增1GW光伏组件、卡本泰克新一代红外线供暖材料制造等项目建设,提速“煤都”向“氢都”和“新能源产业之都”的转变。

三是在科技创新平台上实现重大突破。推动中科院工程热物理研究所大同分所、中科院电工研究所大同基地、同煤集团双创中心、中关村智造大街孵化园、上海漕河泾开发区国际创新创业园、山西能源革命排头兵展示馆等项目建设。打造国内外能源技术创新生态圈,加强与以色列特拉维夫大学、荷兰莱顿大学、德国能源署等国际领先能源研究服务机构互动,在科技创新、技术转移、产学研合作等领域实现突破。依靠绿色金融等手段大力支持科技创新,鼓励和吸引全国、全球的能源技术初创企业落户大同。在储能、氢能、石墨烯、智能电网能源交易中心等关键领域开展战略性布局,抢占能源科技制高点。

四是在深化落实能源体制改革领域取得重大突破。深化电力、煤炭、新能源等领域改革,在“能源市场化交易”和“现代能源治理体系”两个领域形成模式。把握机遇,做好“开发区”和“能源革命科创园”两个国家级增量配电网示范项目,在电力挂牌交易、新能源直供等领域积极探索。把能源降成本作为供给侧改革的重要抓手,继续深化推进大用户直供,切实将能源优势转化为产业优势。

五是在推进园区建设上取得重大突破。“一园一城”是我市争当能源革命尖兵的“牛鼻子”工程,将对前沿技术创新、关键产品示范、创新模式打造、中小企业培育、创新要素聚集起到关键支撑作用。全面加快“一园一城”建设,科创园“23+1”项目全面提速,年内部分工程主体完工,全力打造“中国一流、世界前沿”的能源革命创新园区;“氢都”新能源产业城核心起步区10平方公里的16个新项目全部开工,着力打造智力密集、高技术汇集、新能源企业聚集的高品质产业城。

六是在能源绿色消费和全民共享领域上实现重大突破。聚焦绿色交通、节能建筑、清洁供暖、绿色乡村四大领域,启动规划能源革命“十大乡村”“十大小区”“十大校园”建设规划。打造城市绿色交通网络体系,实施氢燃料公交车和纯电动公交车示范工程,实现加氢制氢运氢一体化。在全市大力推广装配式建筑和被动式超低能耗建筑,开工建设全省首个装配式+被动式超低能耗三星级绿色建筑示范区。创新绿色采暖方式,推动谷电采暖、弃风限光采暖、红外线供暖、空气源/地源热泵供暖等多种方式的优化集成,实现“采煤不见煤”“煤都不烧煤”的历史性转变!

(二)工业大振兴工程

工业是立市之本、富市之要、强市之基。重点抓好工业大振兴五大行动。

一是高端制造大发展行动。要把发展高端制造业放在更加突出位置,围绕高端制造引领,重点推进“陕汽重型汽车百亿产业链”项目,加快陕汽大同公司年产2万辆重卡汽车生产项目开工,抓好重汽大齿公司和北宇专用车公司生产配套技术改造,确保新能源汽车产业链全年产值达到100亿元;大力推进电力机车产业提质增效,加快中车大同公司产能释放,力争年内完成产值65亿元;积极推进北方(大同)军民融合产业园建设,抓好总投资6亿元的国际高端铸造科研试制中心项目的规划启动;全面推进墨西科技、尚镁科技、轻型飞机、北斗显示等高端智能制造项目建设。

二是工业企业大技改行动。突出抓好总投资105亿元的50项重大技改项目,投产达效后年预增产值180亿元。重点推进悦凌空调年产10万套超低温空气源项目,一期工程年内产能达到2万套;重汽大齿公司变速箱及发电机齿轮智能制造项目年内建成投产;国药威奇达中抗青霉素绿色产业链升级项目,年内主体工程完工,一期工程投产达效;同药集团黄芪中药制剂生产线升级改造项目,年内实现部分投产。

三是转型项目大建设行动。突出抓好总投资1188亿元的50项重大新建转型项目,投产达效后年预计增加产值1400多亿元。重点推进同煤60万吨烯烃项目,3月份全面开工,年内部分主体工程完工;中海油年产40亿立方煤制天然气项目,力争上半年开工建设;国网新源浑源150万千瓦抽水蓄能电站项目年内开工。

四是产业集群大培育行动。依托装备制造园区、医药园区、现代纺织园区等产业模式,加快产业集聚,重点培育装备制造、生物医药、现代纺织等产业集群。要立足产业优势,率先在医药产业集群培育上实现新突破,借助同药集团高福院士工作站、国药威奇达和普德药业等企业省级技术中心,加快药品一致性评价和新药品开发,力争年内医药产业产值增长30%以上,并牵头组建全省医药产业联盟集团。

五是现代工业大招商行动。按照《山西省制造业十二大领域发展(招商)图谱》,落实好我市招商引资引智“双十条”和民营经济“二十条”政策,以开发区整合扩区和新获批省级开发区为承载,主动对接京津冀一体化、雄安新区、东南沿海地区产业大转移,大力开展以商招商、产业链招商、特色化招商、区域合作招商等招商活动,着力招商引资推动一批科技含量高、成长性好、带动性强的高新技术产业项目和重大产业转型项目落户我市,持续增强工业大振兴发展后劲。

(三)对外开放大提升工程

把开放落实到项目和载体上,全力争当对外开放“尖兵”,要突出抓好“三个打造”。

一是打造京晋(大同)协作新区。深入贯彻国家支持山西与京津冀加强协作实现联动发展的意见,依托开发区、云州区黄金发展空间,规划面积53平方公里,起步区建设10平方公里,坚持产城融合,突出项目引领,加大基础设施建设的资金配套力度,建设融国际教育、智慧创新、文化创意、医疗康养、国际合作、总部经济、现代物流于一体的现代产业新城。

二是打造对外开放“五大桥头堡”。即中关村智造大街高端制造桥头堡,上海漕河泾开发区长三角东部产业转移项目桥头堡,深圳珠三角高科技产业转移项目桥头堡,以色列特拉维夫大学科技创新成果转化桥头堡,德国新能源产业创造研发桥头堡。在国家战略经济圈和区域经济圈中崭露头角,实现由“挤入”到“纳入”再到“融入”的根本性转折,以此链接产业、链接企业、链接项目,形成大项目好项目成批引进,捆绑进入、梯次建设、滚动发展、滚动投产的工作格局,形成大项目顶天立地、小项目铺天盖地。

三是打造现代交通“三大载体”。启动云冈机场三期改扩建项目建设,新建3.5万平方米候机楼,满足大型飞机起降,逐步向国际机场迈进。成立大同航空产业集团,大力培育低空旅游、航空物流、短途运输、私人飞行等八大业态,打造全省通用航空产业发展示范市;加快完善大同国际陆港功能,启动自贸大厦建设,年内获批保税物流中心。实现航空口岸正式开放。年内开通大同中欧(亚)班列不少于3列。开工建设投资5亿元的晋北国际物流园项目。大张高铁年内开通,集大原高铁项目全面启动,太原至怀仁东动车“五一”开通,年底开至大同高铁南站,确保实现大同至西安动车、高铁全线全贯通!

(四)古城复兴和“五区”联动发展工程

大力推进古城区、御东新区、同煤新区、魏都新区、京晋(大同)协作新区“五区”联动发展,立足未来产业发展方向,推动规划衔接、道路相同、产业互通、基础设施与公共服务共建共享,实现同城化建设、同城化管理、同城化发展。重点抓好四项工作。

一是实施古城复兴工程。古城是大同人民的精神家园,是大同文化的根和灵魂。要坚持把《大同历史文化名城保护规划》作为古城保护和管理的准则,加强历史街巷、古宅老店、建筑风貌和文化元素、民俗风情等的保护。深入挖掘传统优秀文化精华,鼓励和吸引社会资本参与,启动实施府衙、代王府、永兴坊、里坊巷等17个项目,3月1日全部开工,推动百座博物馆建设工程,坚决守住古城文化之魂、大同人民之根。

二是明确“五区”发展定位。按照“分区定位、各具特色、项目拉动、产业互补”的思路,有效整合资源,优化功能结构,提升城市品质。古城区以旅游、商贸等第三产业为主导,打造大同历史文化和古都文化的标识区。御东新区、同煤新区积极承接高端制造、煤基科技、新能源、新材料、新一代信息技术、高端医疗、现代康养、总部经济等战略性新兴产业,打造产业发展新高地。京晋(大同)协作新区主要承接京津冀产业转移和非首都功能疏解,打造对外开放合作先行区。全面启动25平方公里魏都新区规划,深入挖掘传承北魏平城京都文化,带动城市西北片区和新荣区整体发展,打造北魏平城京都文化传承区。

三是完善城市路网。全力推进平城街西跨桥及拥军路片区道路工程建设,年内实施道路工程108公里。加快实施同煤至机场的高架快线工程;加快推进大塘路与御河西路、大塘路与G208国道互通立交工程及御河西路、云冈、环城快速干线的前期准备工作;规划设计第八座跨御河大桥及大同外环高速连接线南延拓展工程,构建大进大出、快进快出的立体化交通体系。

四是优化城市服务功能。巩固行政区划调整成果,理顺城市管理体制,优化城市总体布局,统筹考虑平城、云冈、云州、新荣四区国土空间规划,统一安排基础设施建设,进一步拉大城市建成区发展框架。体育中心、大剧院、美术馆三大场馆年内完工,加快推进会展中心PPP项目。续建永泰门广场,打造华北地区最具活力的“都市大客厅”。建设高铁站前北广场综合枢纽工程以及配套道路工程,新建大同公交客运枢纽,补齐基础设施短板,大力提升城市品质,实现“城市让生活更美好”的追求目标。

(五)乡村振兴工程

坚持把实施乡村振兴战略作为新时代“三农”工作的总抓手,扎实推动农业农村优先发展。重点抓好“五个突出”。

一是突出壮大黄花产业。以云州为核心,以天镇、广灵、灵丘、阳高和浑源等县区为重点,今年力争新增黄花6万亩,总面积达到22万亩,在云州区创建以黄花为主导产业的融合火山森林公园、田园综合体,集种植、加工、科研、产业于一体的大同现代农业经济技术开发区,建设全国优质黄花种植基地、标准化示范区和产品集散中心。

二是突出发展有机旱作农业。毫不动摇抓好粮食生产,确保总产量稳定在16亿斤以上。建设高标准农田19.1万亩。整建制推进灵丘、广灵2县创建市级示范县,灵丘县申报省级示范县,打造3个省级封闭示范片,创建有机旱作农业样板项目区10万亩,推广渗水地膜穴播技术10万亩、旱作节水和地膜减量增效技术15万亩。灵丘、广灵两县要抢抓机遇,大力提升有机旱作农业水平,为全市争创全国有机旱作农业示范市作出积极贡献。

三是突出建设雁门关农牧交错带。完成耕地种草55万亩、粮改饲12万亩,建设阳高正大、云冈四方、天镇中地等10大畜牧标杆园区,提升改造养殖场100个,建设左云、新荣、浑源等8个百万牛羊养殖大县,新增养羊50万只、养牛5万头。严防非洲猪瘟等动物疫情,确保农业生产安全。

四是突出培育农业产业龙头企业。坚持大项目引领,全力抓好农业嘉年华、农副产品物流园区等20个亿元以上项目建设,加快促进北京首农、华润创业、广垦粮油等业内龙头企业项目落户大同。引导农业加快发展城郊农业,促进农林文旅康养产业融合,走出农业+工业+文旅的大同农业转型发展新路。

五是突出改善人居环境。继续实施乡村环境提升工程,下大气力创建示范村200个、整治村1000个,深入开展拆违治乱、农村生活垃圾治理、生活污水处理、厕所革命、卫生乡村建设、清洁能源六大专项行动,使农村环境净起来、绿起来、美起来。

(六)脱贫摘帽攻坚工程

落实乡村振兴战略,聚焦深度贫困,巩固和扩大脱贫成果,确保决战决胜。重点抓好三项工作。

一是攻坚深度贫困。坚持政策向深度贫困聚焦、资金向深度贫困倾斜、项目向深度贫困聚集,“一县一策”,精准帮扶,确保村村有脱贫产业和带动主体,户户有增收项目和脱贫措施,全市实现2.2万贫困人口脱贫,101个贫困村退出,浑源、天镇、广灵三个贫困县摘帽,新荣区贫困村全部退出。

二是解决突出问题。紧盯贫困县退出的14项指标,着力解决易地扶贫搬迁、危房改造、卫生室、饮水安全、道路硬化、文化活动场所和网络覆盖七大突出问题,补齐基础设施和公共服务的短板。天镇、浑源6个易地扶贫搬迁安置点按期竣工,搬迁人口10月底全部入住;危房改造完成2.96万户,天镇、广灵、浑源和新荣四县区实现“危房清零”;91个行政村卫生室达标;200个村、15.2万人饮水安全问题得到全部解决;新建改建农村公路108公里;实现260个行政村综合文化活动场所全达标;建设260个村4G或宽带网络,推进市县电视台节目全覆盖。

三是巩固脱贫成果。坚持责任、政策、帮扶、监管“四不摘”,继续落实已摘帽贫困县、已退出贫困村和脱贫人口的扶持政策。阳高、云州、灵丘、云冈、左云五个县区抓好巩固提升,着力培育后续产业,实现农民持续稳定增收,建立防止返贫保障机制,筑牢返贫防线,让贫困群众健步走上真脱贫、真致富、真小康发展之道。

(七)文旅振兴工程

以文化为灵魂、旅游为载体,突出龙头景区建设,优化“3+3+3+1”发展格局,推动“五环联动”,着力打造国际一流全域旅游目的地。重点抓好五大旅游景区工程。

一是古城文旅业态培育工程。要加大招商引资力度,吸引社会资本参与古城保护,建立多元化投融资机制,为古城发展提供有力支持。以历史文化街区为引领、休闲度假旅游为支柱、文化创意产业为特色,推动古城内业态有机统一,以产业多元化形成古城发展利用新格局,着力打造中国北方集乐宿、美食和文化体验于一体的综合性旅游胜地,让市民在这里有事可做、有商可营、有业可创,再现昔日古都市井繁华。

二是云冈景区大扩容工程。云冈石窟是历史赋予大同的世界级文化艺术宝库。必须站在引领世界文化的高度,对现有景区进行全面扩容升级。紧紧围绕历史价值、宗教文化、艺术魅力,拓展大云冈、大品牌、大市场、大文化,重点开发文化创意、佛学禅修、传统体验等,启动云冈小镇、游客集散中心、文化演艺、传统体验等项目,全面提升云冈石窟的国际影响力,努力将云冈景区打造成感受东西方文化融合的世界级观光胜地。

三是恒山景区大提升工程。恒山是中华五岳之一,悬空寺是世界建筑艺术的瑰宝。要突出围绕景区提质升级和服务重组、产品重构、品牌重塑,全面拓展游客集散体系,调整旅游交通线路,开发文化体验项目,提升服务品质,完善全域景区联动。启动游客集散中心、恒山小镇、温泉开发、世界级恒山森林生态公园等建设,完成景区交通改造、神溪湿地保护和文化体验等项目,努力将恒山景区建设成为弘扬中华文化、感受现代风情的国家级休闲森林公园。

四是长城1号景区大整合工程。大同长城是中华万里长城的重要组成部分,也是全省旅游三大板块的重点区域。要重点围绕空间组合和基础配套,在全面保护的前提下,完成“长城一号”国家风景道建设工程,启动建设汽车营地、艺术基地、长城人家、军事展览、体育运动等项目,积极开发户外休闲、研学度假和风情体验等项目,努力将大同长城建设成为感受历史风韵、体验塞上风情的综合性旅游胜地。

五是火山景区大完善工程。大同火山群是我国六大火山群之一,也是自然赋予大同的重要遗产。要重点围绕功能完善、产品开发和服务提升,进一步加大招商引资力度,加强战略投资合作,全面完善景区旅游服务功能,重点建设好集散中心、地质博物馆和景区导览系统等,大力培育住宿、餐饮、娱乐等业态开发,努力将大同火山群打造成为京津冀游客亲近自然、感受神奇、体验快乐的综合旅游胜地。

同时,进一步开发平型关、桑干河、土林、晋华宫、温泉、冰雪旅游,着力完善公共服务体系,加速智慧旅游系统,提升全域旅游品质,丰富大同冬季旅游项目和内容,形成功能完备、四季宜游的发展格局。

(八)生态文明建设工程

统筹山水林田湖草系统治理,加快建立健全生态环境治理体系,以壮士断腕的决心、背水一战的勇气、攻城拔寨的拼劲,做好生态保护的加法和污染排放的减法,落实好中央和省环保督察整改事项,坚决打赢污染防治攻坚的人民战争。重点抓好三大攻坚战。

一是坚决打赢蓝天保卫战。倍加珍惜来之不易的“大同蓝”,坚持转型、治企、减煤、控车、降尘“五管齐下”,坚决整治“散乱污”企业,持续推动清洁取暖和散煤替代由城市建成区向农村扩展,实现建筑工地绿色施工全覆盖,确保今年空气质量再提升,确保年内二级以上良好天气达到300天以上。

二是持续打好碧水保卫战。统筹实施“五水共治”,加快推进御河、十里河等重点流域生态修复工程,实施东郊污水处理厂搬迁和西郊污水处理厂提标扩容工程,深入开展入河排污口整治工作,加大截污纳管力度,继续补齐城镇污水收集和处理设施短板,新建和改造城市污水管线60公里,全力确保固定桥、利仁皂两个国考断面水质高标准退出劣五类。做好生态调水工作,增强河流纳污能力,国庆节期间御河南北20公里河道全线注水;加快建成御河两岸七大片区沿河公园和一个湿地公园,提升“一河两岸”人居环境和城市景观,着力打造风景如画的生态环境。

三是统筹打好净土保卫战。完成农用地、重点行业企业用地土壤污染状况调查,实施农用地分类管理和建设用地准入管理。启动土壤环境基础数据库和信息化管理平台建设。加强农业面源污染防控,继续在阳高县开展地膜回收试点工作,完成全市74个畜禽养殖场粪污治理和资源化利用,大力推进“开源一号”治理修复项目和云冈、天镇、灵丘农用地超标区域治理修复工程。召开“万人上恒山,生态大治理”誓师大会,高质量做好6.2万亩浑源矿山整治生态修复工程,今年首先完成大磁窑到官王铺道路带、阳光煤业修复示范点、多个露天采区“一带一点多区”修复工程,面积达到3.17万亩。在全市范围内同步开展生态破坏大排查大整治行动,守住生态文明底线,守住绿水青山。

(九)财源培育壮大工程

培植财源,开源节流,加强征管,为全市转型发展和高质量发展提供有力支撑。要实施三大培植工程。

一是发展经济,培植财源。在做大做强工业经济的基础上,加快发展一产、三产,延长产业链,拓展价值链。扶持发展民营经济,落实省鼓励和支持民营企业发展“30条”、市鼓励和支持民营企业发展“20条”政策措施,培育50家中小微企业进入规模工业企业,把更多的民营企业扶持成“税收大户”,成为支撑全市经济的“主力军”。培育壮大县域经济,坚持“因地制宜、错位发展、一县一业、整体提升”的思路,精心培育发展风电光电等能源产业、黄花黄芪等兴农产业、康养旅游等现代产业,着力补齐县域经济发展短板,尽快走出各具特色的富民兴县发展之路。

二是多措并举,开源节流。密切关注国家和省投资政策和动态,积极争取上级项目和资金支持;梳理公益性项目,多争取政府债券资金支持。加强财政资金引导,“四两拨千金”,有力撬动社会资本。加大土地收储出让力度,努力增加土地收入。推动盘活城市国有资产,实现保值增值。推进国有资产证券化,加快供排水集团股份制改造和上市,实现资本利润最大化。市县财政一律按照不低于10%的比例压减一般性财政支出,“三公”经费只减不增,降低行政运行成本,把有限的资金用在“刀刃”上。严格预算管理,加快支出进度,加强绩效管理,提高财政资金使用效益。

三是加强征管,应收尽收。加强重点行业、企业税收征管,加大政府住房基金和“两权价款”等非税收入征收管理,强化税费稽查,做到应收尽收。

(十)重点领域改革工程

以“改革关头勇者胜”的魄力,加快重点领域和关键环节体制机制改革,着力构建四梁八柱,充分释放改革红利。

一是深化开发区改革。以京晋(大同)协作新区为牵引,全面深化开发区“三化三制”改革,重点抓好土地审批、人才引进、项目建设等重大配套改革,充分发挥政策叠加效应。加快推动开发区“专业化、市场化、国际化”的推进力度,在全国叫响大同开发区的投资环境品牌,着力打造引领转型的尖刀班、突击连。

二是深化国资国企改革。全面加强国有资产运营管理,提高国有资产使用效益。大力发展混合所有制经济,积极引导民营企业参与市属企业改制重组,支持国有控股企业以多种方式入股非国有企业。推动“僵尸企业”依法破产出清,为下一步盘活资源、实现资产再利用扫清道路。加快剥离国有企业办社会职能,确保驻同27户央企和省属企业“三供一业”分离移交工作稳步推进,确保企业瘦身健体、聚焦主业、轻装前行。

三是深化“放管服效”改革。加快行政审批制度改革,进一步优化行政运行机制;深化承诺制改革,试行“容缺后补”,探索“一颗印章管审批”,推行审批服务“一窗通办”和政务服务“网上办理”。大力开展“五送”优质服务,营造“六最”营商环境,坚决消除“卷帘门”“玻璃门”“旋转门”现象,让不找人办事成为一种习惯。

(十一)重大风险防范工程

准确把握改革发展稳定面临的新情况新问题新挑战,坚持底线思维,保持斗争精神,有效防范化解重大风险。一是在全市范围内开展风险隐患大排查,建立“问题清单”“落实清单”“责任清单”,增强防范化解风险的针对性、有效性,把风险防范抓在早、控制在小、化解在源头、消灭在萌芽。二是稳妥推进政府隐性债务存量化解,做到坚定、可控、有序、适度;加强政府债务预算管理和限额管理,规范政府举债融资行为,坚决遏制隐性债务增量,全市综合债务率控制在风险预警线之内,风险债务率指标逐步改善。三是强化政府金融监管和服务能力,完善风险监测预警机制,加强金融风险源头管控,有序推进互联网金融风险专项整治,妥善处置非法集资、互联网金融、重点企业债务等金融风险,守住不发生区域性金融风险的底线。四是坚决防范社会风险,深入推进扫黑除恶专项斗争,在打防并举、标本兼治上下功夫;实施“雪亮工程”,完善社会治安技防体系。坚持发展新时代“枫桥经验”,扎实开展矛盾纠纷大排查、隐患大整改,防止各类矛盾叠加集聚,从源头上提升维护社会稳定能力和水平,增强人民群众安全感。

(十二)民生事业改善工程

坚持以人民为中心的发展思想,办更多的民生实事,把财富蛋糕更多地分给群众,让他们充分感受到民生清单的厚重温暖。

一是加快社会事业发展。优先发展教育事业,推进乡镇寄宿制学校建设,提高义务教育城乡一体化发展水平,打造义务教育优质均衡示范区;不断改善高中办学条件,提升高中阶段教育水平;持续规范治理校外培训;整合职业教育资源,加快高等职业院校建设,深入开展现代学徒制试点,推进校企合作、产教融合。深化市厅共建,扶持办好大同大学。深化卫生体制改革,推进优质医疗资源共享,加快完善城乡医疗服务体系,发展繁荣中医药事业,全面提高医疗卫生水平。加快省级文明城市创建步伐,促进城市环境、品位和市民素质整体提升,切实增强市民的归属感和幸福感;积极回应群众关切,在全长25公里的御河东岸,新建篮球、足球、门球等各类群众性文体设施和场馆。承办好全国第二届青年运动会夏季赛事,推动全民健身和竞技体育事业全面发展。

二是加大社会保障力度。全面做好就业创业工作,努力让每一个人“人尽其才”;完善覆盖城乡的社会保障体系,逐步提高城乡居民基本医疗、大病救助、低保五保水平,实现“应保尽保”;加强对老年人的关心关爱,健全完善养老服务体系,加强城乡养老服务设施建设,推动居家和社区养老服务改革,确保“老有颐养”。不断提升住房保障水平,筹集棚改安置房房源15000套,年内新开工建设1800套,全力推进棚改PPP项目建设运营工作,让各类群体都能有房住、住好房、能安居。积极落实优抚政策,加强退役军人服务管理工作,让最可爱的人得到全社会的尊崇。

三是加强社会治理创新。健全社区基础设施,强化社区服务功能,推进政府向社会力量购买服务,持续开展“三社联动”,提升城乡社区治理水平。强化应急管理工作,提升防灾减灾救灾能力。强化安全生产责任落实,坚决杜绝重特大安全事故发生,推进城市安全发展。加强国防动员、“双拥”创建等工作,推动军民融合发展。规范宗教事务管理,提升宗教工作法治化水平。

三、全面加强政府自身建设

各位代表,大同正处在负重爬坡、滚石上山的攻坚期,形势逼人、不进则退。站在新的历史起点上,直面全市人民的新期待,我们将以“改革创新、奋发有为”大讨论牵引全年政府工作的新开局,全面有效提振政府系统广大干部干事创业的精气神,努力打造勤政为民、务实高效、清正廉洁、人民满意的政府,为圆满完成全年目标任务、在“两转”基础上全面拓展新局面提供坚强保障。

一是尊崇党章,严守纪律规矩。始终把政治建设摆在首位,牢固树立“四个意识”,不断增强“四个自信”,坚决做到“两个维护”,政府一切工作都要与以习近平同志为核心的党中央高度看齐,做到政治上旗帜鲜明、思想上同心同向、行动上紧紧跟随,确保习近平总书记视察山西重要讲话精神在大同深深扎根、开花结果。要及时与省委市委重大决策部署对表对标,确保大同经济建设和各项社会事业始终立足较高政治站位、保持正确方向、拥有开阔视野、不断破浪前行。

二是依法行政,维护宪法权威。主动接受市人大及其常委会的法律监督、工作监督和市政协的民主监督,认真办理人大代表建议和政协委员提案。认真落实《监察法》,推进公职人员依法行政、依法履职。加强重点领域执法监督检查,大力推进政务公开,广泛接受公众监督和社会舆论监督,确保权力在阳光下运行。严格落实“三重一大”制度,充分吸纳各方面意见和建议,切实提高政府谋大事、抓实事、办好事的能力和水平。加快推进政府机构改革,着力构建办事高效、运转协调、行为规范的行政管理体系。

三是锐意改革,激发发展活力。扎实开展“改革创新、奋发有为”大讨论,对标“6个破除”“6个着力”“6个坚持”,坚决破除僵化保守、因循守旧、封闭狭隘、资源依赖、随遇而安、慵懒散漫,着力解决改革意识不强、创新精神不足、扩大开放不够、市场理念不浓、工作标杆不高、作风不硬不实等突出问题,坚持以改革破解发展难题、以创新激发动力活力、以开放促改革发展、以市场打开发展新通途、以一流标准创造一流业绩、以过硬作风彰显担当作为。要高标准、严要求、硬考核,科学建立《大同市政府工作标准体系》《大同市政府工作推进办法》,大幅度抬升工作标杆,实施高效能、科学化、项目式管理,提升干部干事创业的执行力,推动政府各项工作提质增效,确保实现一季度“开门红”、半年“双过半”、全年任务圆满收官。

四是担当作为,勇创一流业绩。勇于破除资源依赖的“挤出效应”,大力弘扬市场意识和创新精神,在争当能源革命和对外开放“两个尖兵”上敢闯敢为,在推进“三大振兴”上大胆作为,在实施“三大攻坚战”上奋发有为,确保“大同”号列车在奋进新时代的历史航程上敢于抢点、永不晚点、频现亮点,时时冲锋在前、站站大有收获。要始终保持勇立潮头不让、把住红旗不放的胆气和魄力,敢与强者比、敢与能者赛,勇攀经济高质量发展的顶峰,力克能源革命重大突破的堡垒,强攻贫中之贫、困中之困、难中之难、坚中之坚的硬坎,确保大同各项工作再上新台阶、再创新辉煌。

五是转变作风,狠抓工作落实。始终坚持激情干事、精准干事、开拓干事,抓住每一天、抓实每一月,撸起袖子加油干,干就干到最好,争就争到第一,以责任和务实安排日程,用心血和汗水浇灌行程,一锤接着一锤敲,一件接着一件办,把钉钉子精神写在大同大地上。要充分发扬求真务实精神,坚持百姓为本、问计于民,干部带头、取信于民,实干苦干、普惠于民,推动政府工作不断往深里走、往实里做。要主动深入基层、深入一线、深入群众,多与群众加强来往、联络感情、沟通思想,确保政府工作上接天线、下接地气。要从严建立督查考核、责任清单和群众评议制度,倒逼各级各部门牢固树立责任意识、指标意识、完成意识,推动政府工作干一件、成一件,向全市人民交出优秀答卷。

六是恪尽职守,打造廉洁政府。扛起“两个责任”,落实“一岗双责”,持之以恒抓好政府系统党风廉政建设和反腐败工作。充分发挥审计监督作用,加大审计发现问题整改力度,做到应审尽审、凡审必严,失职问责、严肃追责,努力构建集中统一、全面覆盖、权威高效的审计监督体系。严格控制“三公”经费,勒紧裤带过日子,把该省的钱坚决省下来,尽最大可能让位于经济发展和民生改善。严格落实中央八项规定精神,驰而不息纠正“四风”,坚决执行《准则》《条例》,努力争取“两转”基础上的更大进步。

各位代表,历史不能往复,现在必须把握,未来亟待开创。让我们高举习近平新时代中国特色社会主义思想伟大旗帜,在省委省政府和市委的正确领导下,负重加压、砥砺前行,改革创新、奋发有为,为成功走出资源型城市转型升级新路、为加快建设美丽富裕幸福大同、为夺取全面建成小康社会的决定性胜利而努力奋斗!
